# Comparison of MRI, PET, and 18F-choline PET/MRI in patients with oligometastatic recurrent prostate cancer

**DOI:** 10.1007/s00261-021-03131-7

**Published:** 2021-05-28

**Authors:** Laura Evangelista, Gianluca Cassarino, Alberto Lauro, Alessandro Morlacco, Matteo Sepulcri, Alex Ahn Li Nguyen, Francesco Ietto, Diego Cecchin, Carmelo Lacognata, Pietro Zucchetta

**Affiliations:** 1 Nuclear Medicine Unit, Department of Medicine (DIMED), University of Padova, Via Giustiniani, 2, 35128 Padua, Italy; 2 Radiology Unit, University-Hospital of Padova, Padua, Italy; 3 Department of Surgical Oncological and Gastroenterological Sciences, Urology University of Padua, Padua, Italy; 4 Radiotherapy Oncology Unit, Veneto Institute of Oncology IOV – IRCCS, Padova, Italy

**Keywords:** Prostate cancer, Positron emission tomography, Magnetic resonance imaging, Choline, Oligometastatic disease

## Abstract

**Objectives:**

The aims of the study were (i) to examine the PCa detection rate of 18F-choline (FCH) PET/MRI and (ii) to assess the impact of PET/MRI findings in patients with PCa who develop OMD using PSA response as a biomarker.

**Methods:**

We retrospectively analyzed a cohort of 103 patients undergoing FCH PET/MRI for biochemical recurrence of PCa. The inclusion criteria were (1) previous radical prostatectomy (RP) with or without adjuvant radiotherapy (RT); (2) PSA levels available at the time of PET; (3) OMD, defined as a maximum of 5 lesions on PET/MRI; and (4) follow-up data available for at least 6 months after PET. All images were reviewed by two nuclear medicine physicians and interpreted with the support of two radiologists.

**Results:**

Seventy patients were eligible for the study: 52 patients had a positive FCH PET/MRI and 18 had a negative scan. The overall PCa detection rates for MRI, PET, and PET/MRI were 65.7%, 37.1%, and 74.3%, respectively. Thirty-five patients were treated with radiotherapy (RT), 16 received hormonal therapy (HT), 3 had a combined therapy (RT + HT), and 16 (23%) underwent PSA surveillance. At follow-up, PSA levels decreased in 51 patients (73%), most of whom had been treated with RT or RT + HT. Therapeutic management was guided by PET/MRI in 74% of patients, which performed better than MRI alone (68% of patients).

**Conclusion:**

FCH PET/MRI has a higher detection rate than MRI or PET alone for PCa patients with OMD and PSA levels > 0.5 ng/mL, prompting a better choice of treatment.

**Supplementary Information:**

The online version contains supplementary material available at 10.1007/s00261-021-03131-7.

## Introduction

Prostate cancer (PCa) is the most commonly diagnosed malignant disease in the adult male population of Europe and North America [1]. The primary treatments most often used are radical prostatectomy (RP) or radiotherapy (RT), either external beam RT (EBRT) or brachytherapy.

Biochemical recurrences (BCR) of PCa occur in 27–53% of patients treated with RP or RT [2], depending on various risk factors, such as a high Gleason score (GS), a short PSA doubling time (PSAdt), and status of surgical margins or locally advanced disease [3–5]. In patients with a BCR, there is no consensus on the optimal imaging modality for detecting small local recurrences in cases with low PSA levels. Conventional imaging, i.e., contrast-enhanced computed tomography (CT), and bone scans are not sensitive enough. Multiparametric MRI (mpMRI) with high soft tissue contrast has a sensitivity of 90% for the identification of local recurrence in case of PSA levels inferior to 1 ng/ml [6].

In the restaging scenario, independently from the PSA levels, positron emission tomography (PET)/CT with 11C/18F-Choline, 18F-Fluciclovine, and 68 Ga-PSMA has shown a sensitivity of 86%, 86%, and 76% and specificity of 93%, 76%, and 45% [7–9], respectively. The overall detection rate of PET/CT decreases significantly, however, in the case of low PSA levels.

The introduction of PET/MRI scanners makes it possible to combine the molecular information obtained by PET with the high soft tissue contrast and the functional information afforded by MRI. The increasing clinical use of modern imaging techniques has improved the detection of low-volume metastatic disease, prompting the definition of a new clinical entity—oligometastatic disease (OMD). Identifying the extent and location of metastases and distinguishing between oligo- and poly-metastatic disease have important implications for patient management, influencing the choice of treatment [10, 11].

The aims of this study were to (i) examine the detection rate of 18F-choline (FCH) PET/MRI in patients with BCR after RP and (ii) assess the impact of PET/MRI findings in PCa patients with OMD using PSA response as a biomarker.

## Materials and methods

### Study population

We retrospectively analyzed a cohort of 103 patients undergoing FCH PET/MRI for BCR (defined as PSA levels higher than 0.2 ng/mL in 2 consecutive assays and rising) from May 2017 to July 2020. Our inclusion criteria were (1) previous RP with or without adjuvant RT, (2) a PSA level available at the time of PET, (3) OMD defined as a maximum of 5 lesions on PET/MRI scans, and (4) follow-up data available for at least 6 months after PET. Patients undergoing hormonal therapy (HT) at the time of PET and those given RT as primary treatment were excluded. All patients gave their informed consent before undergoing PET/MRI. The study was approved by the local ethical committee and was performed in accordance with the Declaration of Helsinki.

### PET/MRI protocol

PET/MRI was acquired using hybrid equipment (Biograph mMR®; Siemens Healthcare, Erlangen, Germany) with 3-T MRI. FCH was administered intravenously (at a dose of 3 MBq/kg of body weight). PET study of the pelvis was conducted for 30 min while simultaneously acquiring T2-weighted turbo spin-echo, T1-vibe after contrast enhancement, T2-haste, T1-vibe fat-saturated, and diffusion-weighted imaging (DWI b50, 800, and 1400) sequences for the MRI component. Following a single dose (0.1 mmol/kg) of gadobutrol (Gadavist, Bayer Healthcare, Berlin, Germany), 7 phases were acquired sequentially with a 26-s temporal resolution. Then total-body PET images were acquired using a 3-min-per-bed protocol with the simultaneous acquisition of T2-haste, T1-vibe fat-saturated (3 mm thick), and DWI sequences (b50 and 1000). A T1-vibe sequence (2 mm thick) was used for the lung scan.

MR-based attenuation correction maps (MRAC) were calculated using a 4-class segmentation technique [12]*.*

### Image analysis

All images were reviewed by two nuclear medicine physicians and two radiologists with at least 10 years of experience with PET and MRI, respectively, using dedicated software (Syngo.via, Siemens Healthcare, Erlangen, Germany). PET/MRI scans were interpreted as follows: PET was defined as positive in the presence of an FCH uptake greater than that of the background activity, excluding foci of physiological activity. MRI was considered positive in the case of an increased enhancement after administering the contrast agent and subsequent rapid wash-out in the MRI sequences or a significantly restricted diffusion in the DWI sequences. At MRI, local recurrence after radical prostatectomy was defined in the presence of a focal or nodular area in the surgical bed that demonstrates (1) low signal intensity on T2-weighted images, (2) restricted diffusion on diffusion-weighted images, and (3) early arterial enhancement in tumor nodule with venous washout avid enhancement on dynamic contrast-enhanced MRI [13, 14].

The RECIST 1.1 criteria were used to interpret the MRI [15], in particular for lymph node and bone recurrences. PET/MRI was considered positive in patients found positive on PET or MRI, or both.

All imaging studies were classified in terms of diagnostic accuracy using the following criteria:

- true positive (TP), patients with evidence of recurrence on imaging and PSA levels decreasing or remaining stable after therapy (considered as evidence of a biochemical response), or increasing PSA levels in patients under clinical surveillance;
- true negative (TN), patients with no evidence of recurrence on imaging and decreasing or stable PSA levels without any therapy;
- false positive (FP), patients with evidence of recurrence on imaging and decreasing or stable PSA levels without any therapy;
- false negative (FN), patients with no evidence of recurrence on imaging and rising PSA levels without any therapy, or despite starting therapy, or with declining PSA levels after therapy.

### Statistical analysis

Categorical variables are expressed as numbers and percentages and continuous variables as medians and ranges. The Mann–Whitney test was used to assess the association between PSA levels and MRI, PET, and PET/MRI results, by patient and site analyzed. PET, MRI, and PET/MRI detection rates were computed. Diagnostic accuracy was calculated using the standard method, considering sensitivity, specificity, positive and negative predictive values, and accuracy. A *p* value < 0.05 was considered statistically significant. The statistical analysis was performed with the SPSS software for Windows v.19 (SPSS Inc., Chicago, IL, USA).

## Results

**Fig. 1:**
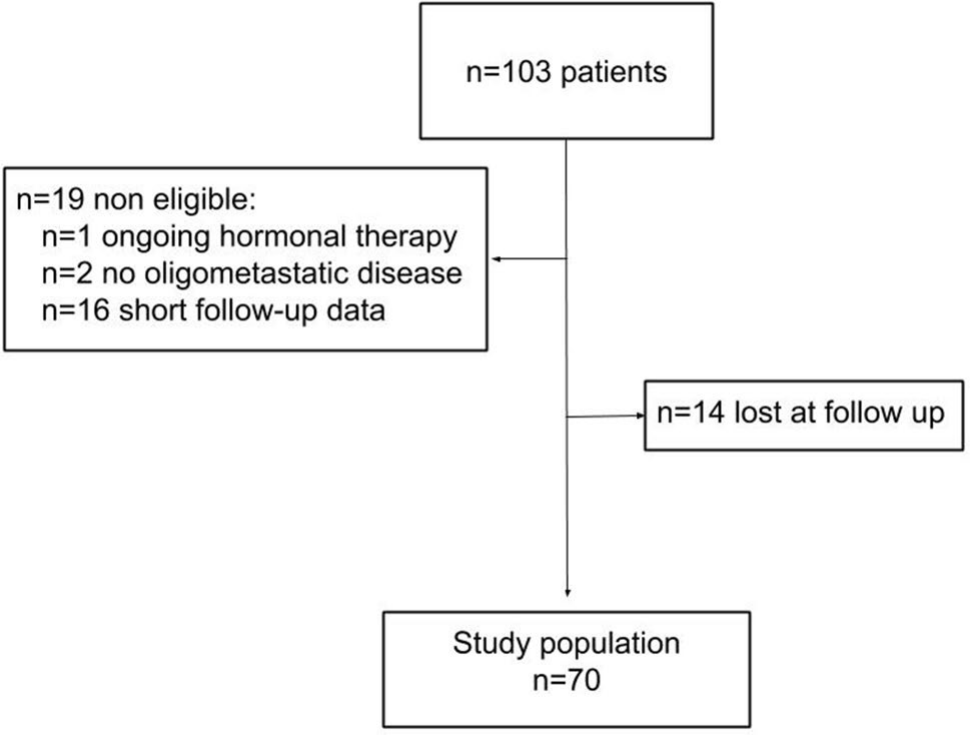
Flow chart of the patient population selected

Out of an initial sample of 103 patients, 70 (68%) patients were eligible for the study because 19 did not meet our inclusion criteria, and complete follow-up data were lacking for 14 patients (Fig. 1). Table 1 shows the patients’ characteristics.

**Table 1:** Characteristics of patients

Variables	
Median age (range), years	71 (54–86)
Gleason score, *n* (%)	
≦ 6	11 (15.7)
= 7	25 (35.7)
> 7	30 (42.9)
N/A	4 (5.7)
Surgery, *n* (%)	
No	–
Yes	70 (100)
Lymphadenectomy, *n* (%)	
No	33 (47.1)
Yes	37 (52.9)
Margins Status, *n* (%)	
No	21 (30)
Yes	10 (14.3)
N/A	39 (55.7)
Stage, *n* (%)	
I	8 (11.4)
II	16 (22.9)
III	31 (44.3)
IV	3 (4.3)
N/A	12 (17.1)
Radiotherapy, *n* (%)	
No	60 (85.7)
Yes	10 (14.3)
Hormonal therapy, *n* (%)	
No	60 (85.7)
Yes	10 (14.3)
Hormonal therapy at PET time, *n* (%)	
No	70 (100)
Yes	–
Median time between surgery and PET (range), in years	9 (1–20)
Median PSA at PET time (range), in ng/mL	0.49 (0.1–5.60)
PSA at PET time category, *n* (%)	
< 0.5 ng/mL	36 (51.4)
0.5–1.0 ng/mL	23 (32.9)
1.1–2.0 ng/mL	6 (8.6)
> 2.1 ng/mL	5 (7.1)

*N/A* not available

Forty-six patients had a positive MRI and 26 had a positive PET scan. Median PSA levels were significantly higher in patients with a positive MRI scan (0.29 ng/mL vs. 0.59 ng/mL; *p* < 0.01), while the difference in PSA levels between patients with and without positive PET results was not statistically significant (0.44 ng/mL vs. 0.61 ng/mL, for negative and positive PET; *p* = 0.127). Fifty-two patients had positive PET/MRI findings. The median (range) PSA levels in patients with a negative and positive PET/MRI were 0.28 ng/ml (0.1–1.80) and 0.63 ng/ml (0.13–5.60), respectively (*p* < 0.001).

The overall detection rates for MRI, PET, and PET/MRI were 65.7%, 37.1%, and 74.3%, respectively. The corresponding detection rates for MRI, PET, and PET/MRI based on site of recurrence were 62.9%, 18.6%, and 62.9% in the prostatic fossa; 8.6%, 18.6%, and 20% in the lymph nodes; 5.7%, 4.3%, and 5.7% in distant organs. The numbers of lesions detected were 55, 31, and 63, respectively, for MRI, PET, and PET/MRI.

**Fig. 2:**
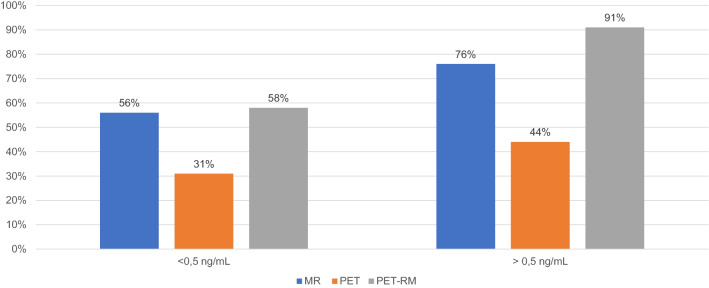
Distribution of detection rates for MRI, PET, and PET/MRI by PSA levels

**Fig. 3:**
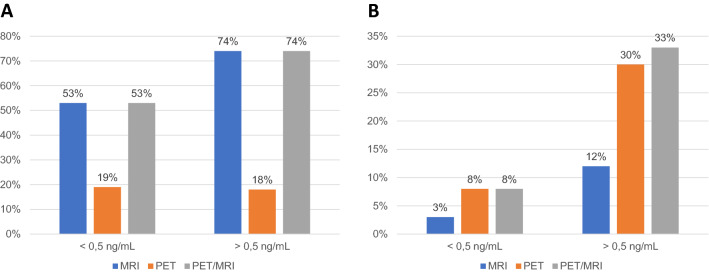
Distribution of detection rates for MRI, PET, and PET/MRI, by site of recurrence, and PSA levels (**a** PSA < 0.5 ng/mL and **b** PSA > 0.5 ng/mL)

Based on PSA levels, the detection rates for MRI, PET, and PET/MRI were 56%, 31%, and 58%, respectively, in patients with PSA levels < 0.5 ng/mL, as opposed to 76%, 44%, and 91% in those with PSA levels > 0.5 ng/mL (Fig. 2; Table 1s). In the subset of patients with PSA levels < 0.5 ng/mL, images suggestive of local recurrence were seen in 53%, 19%, and 53% of cases, respectively, for MRI, PET, and PET/MRI. In the subset of patients with PSA levels > 0.5 ng/mL, this applied to 74%, 18%, and 74% of cases (Fig. 3A; Table 2s). Similarly, suspected recurrent lymph node metastases were detected on MRI, PET, and PET/MRI in 3%, 8%, and 8% versus 12%, 30%, and 33% of patients with PSA levels < or > 0.5 ng/mL, respectively (Fig. 3B; Table 2).

**Table 2:** Distribution of imaging results, in accordance with the site-based analysis, the clinical variables, and follow-up data

PSA value	MRI				PET				PET/MRI			
	Neg. fossae (*n* = 26)	Pos. fossae (*n* = 44)	Neg. LN (*n* = 64)	Pos. LN (*n* = 6)	Neg. fossae (*n* = 57)	Pos. fossae (*n* = 13)	Neg. LN (*n* = 57)	Pos. LN (*n* = 13)	Neg. fossae (*n* = 26)	Pos. fossae (*n* = 44)	Neg. LN (*n* = 56)	Pos. LN (*n* = 14)
< 0.5 ng/mL 0.5–1.0 ng/mL 1.1–2.0 ng/mL > 2.1 ng/mL	17(47) 8(35) 1(17) 0	19(53) 15(65) 5 (83) 5(100)	35(97) 20(87) 5(83) 4(80)	1(3) 3(13) 1(17) 1(20)	29(81) 20(87) 5(83) 3(60)	7(19) 3(13) 1(17) 2(40)	33(92) 15(65) 5(83) 4(80)	3(8) 8(35) 1(17) 1(20)	17(47) 8(35) 1(17) 0	19(53) 15(65) 5(83) 5(100)	33(92) 14(61) 5(83) 4(80)	3(8) 9(39) 1(17) 1(20)
GS < = 6 GS = 7 GS > 7	2(18) 8(32) 16(53)	9(82) 17(68) 14(47)	11(100) 23(92) 26(87)	0 2(8) 4(13)	8(73) 20(80) 26(87)	3(27) 5(20) 4(13)	10(91) 20(80) 23(77)	1(9) 5(20) 7(23)	2(18) 8(32) 16(53)	9(82) 17(68) 14(47)	10(91) 20(80) 22(73)	1(9) 5(20) 8(27)
No PSA decline PSA decline Stable PSA^a^	9(47) 15(35) 2(25)	10(53) 28(65) 6(75)	17(89) 40(93) 7(88)	2(11) 3(7) 1(12)	18(95) 33(77) 6(75)	1(5) 10(23) 2(25)	13(68) 37(86) 7(88)	6(32) 6(14) 1(12)	9(47) 15(35) 2(25)	10(53) 28(65) 6(75)	13(68) 36(84) 7(88)	6(32) 7(16) 1(12)

The results are expressed as number (percentage)

*GS* gleason score, *PSA* prostate-specific antigen, *Neg*. Negative, *Pos*. Positive, *LN* lymph node

^a^Variation in PSA value between 0 and 0.1 ng/ml

**Fig. 4:**
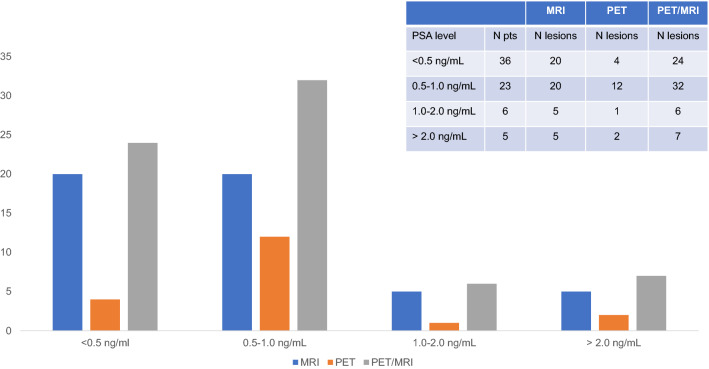
Distribution of detection rates for MRI, PET, and PET/MRI, by number of lesions, and PSA levels (**a** PSA < 0.5 ng/mL and **b** PSA > 0.5 ng/mL)

At lesion-based analysis, 18F-Choline PET/MRI was able to identify a larger number of lesions as compared to PET and MRI alone (Fig. 4). Based on the PSA levels and the number of enrolled patients, PET/MRI showed a high lesion detection rate for a PSA ranging between 0.5 and 1 ng/mL.

After PET/MRI, 16 patients (23%) were followed up without any therapy, 35 (50%) underwent RT alone, 16 (23%) had HT, and 3 (4%) had combined treatments (RT +HT). During the follow-up (median time from PET to latest PSA assay 9 months [range 6–31 months]), PSA levels decreased in 43 (61.4%) patients, remained stable (it means varying between 0 and 0.1 ng/mL) in 8 patients [11, 4], and not reducing in the residual 19 (27.1%).

In particular, PSA levels decreased or remained stable in 31/38 (61%) patients given RT or RT + HT. In this subset of 31 patients, PET/MRI detected more lesions than MRI alone or PET alone (75% vs. 69% and 35%, respectively). PET/MRI therefore identified 6% and 40% more responders to RT or RT + HT (in terms of PSA levels) than MRI or PET, respectively. In fact, PET/MRI prompted changes to the definition of the clinical target volume (CTV) and RT fields in 9 out of 22 patients (41%) with available RT planning data. These changes involved the inclusion of a simultaneous integrated boost (SIB) on recurrences in the prostatic bed during salvage RT and stereotactic RT treatment (SBRT) of positive nodes or bones (Table 2s).

In the whole study population (*n* = 70), appropriate therapeutic management was guided by PET/MRI in 74% of patients, and PET/MRI performed better than MRI alone (68% of patients) for treatment decision-making purposes.

**Fig.5:**
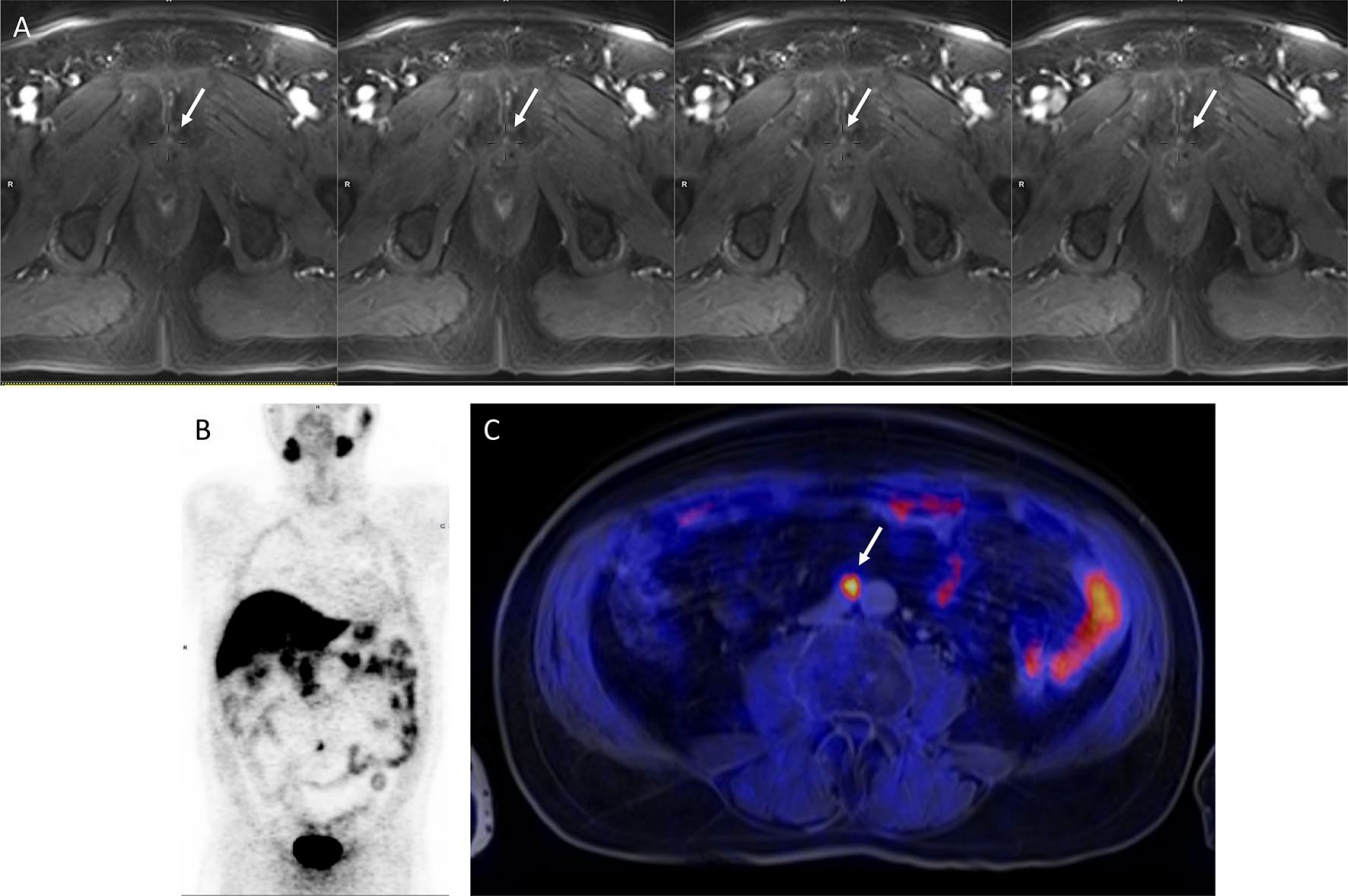
A 73-year-old man with a biochemical recurrence of prostate cancer (PSA = 2.46 ng/mL), treated with radical prostatectomy and pelvic lymph node dissection (pT3N0M0, Gleason Score: 9).**a** Magnetic resonance images showed the presence of an enhanced lesion in the prostatic fossae (arrow), compatible with a local recurrence. At PET (**b**, maximum intensity projection, MIP) and PET/MRI images (**c**), a focal uptake of FCH was found in a right lumbar lymph node (arrow), compatible with distant metastases

Table 3 shows the diagnostic accuracy of PET/MRI, MRI, and PET. It emerged that PET/MRI was more sensitive than MRI or PET alone. On the other hand, MRI proved more specific than PET/MRI or PET, due to a relatively smaller number of false positives. This latter finding can be correlated with the presence of inflammation, which can accumulate FCH at the site of a potential PCa recurrence. In Fig. 5 is reported an example of a FCH PET/MRI positive scan.

**Table 3:** Diagnostic accuracies for PET/MRI, MRI, and PET alone

	TP	TN	FP	FN	Sensitivity (CI 95%)	Specificity (CI 95%)	PPV (CI 95%)	NPV (CI 95%)	Accuracy (CI 95%)
PET/MRI	49	5	3	13	79% (69–89%)	63% (29–96%)	94% (88–100%)	28% (3–59%)	77% (67–87%)
MRI	44	6	1	19	70% (59–81%)	86% (60–100%)	98% (94–100%)	24% (7–55%)	71% (61–82%)
PET	24	5	2	39	38% (26–50%)	71% (38–100%)	92% (86–99%)	11% (0–35%)	41% (30–53%)

*CI* confidence interval, *TP* true positive, *TN* true negative, *FP* false positive, *FN* false negative, *PPV* positive predictive value, *NPV* negative predictive value

## Discussion

In the present study, we found a higher PCa recurrence detection rate for FCH PET/MRI than for PET and MRI alone (74.3% vs. 37.1% and 65.7%, respectively), particularly in patients with PSA levels > 0.5 ng/mL—though an acceptable detection rate was also achieved in patients with very low PSA levels (58%).

Previous publications reported on overall detection rates for FCH or 11C-choline PET/MRI in recurrent PCa [2, 16–18]. For instance, Eiber et al. [16] found an overall detection rate of 84% in 75 patients with median PSA levels of 2.6 ng/mL (range 0.2–88 ng/mL). In 58 patients, Achard et al. [17] found an overall positivity rate for the FCH hybrid PET/MRI of 58.6%, while it was 44% among patients with PSA levels ≤ 2 ng/ml and 12.5% for those with PSA levels < 0.5 ng/mL. Similar results were reported by Riola Parada et al. [2] who found an overall detection rate of 55.56% for FCH PET/MRI in 27 patients with a median PSA level of 2.94 ng/mL (range 0.18–10 ng/mL). In our experience, the overall detection rate for PET/MRI was 74% in 70 patients with an early biochemical PCa recurrence and a median PSA level of 0.49 ng/mL (range 0.1–5.60 ng/mL). There may be various reasons for the different detection rates obtained in the above-mentioned studies, including (1) the study population selected (i.e., type of treatment before or at the time of PET, characteristics of the primary tumor, and so on), (2) different criteria used to interpret PET/MRI findings, (3) the number/type of MRI sequences used, and (4) the image reader’s experience.

The recent introduction of radiolabeled PSMA has significantly improved the detection rate in recurrent PCa, especially for lower PSA levels. Several experiences with 68 Ga-PSMA PET/MRI have now been published [19, 20]. Kranzbuhler et al. [19] reported an overall detection rate of 78.6%, 24%, and 76%, respectively, for 68 Ga-PSMA-11 PET/MRI, mpMRI, and PET in a population of 56 patients with a BCR of PCa after RP (median PSA: 0.99 ng/mL). The authors found a higher detection rate for higher PSA levels, reaching 72.7% for PSA < 0.5 ng/mL. They underscored the additional value of PET/MRI in detecting metastatic lymph nodes rather than local recurrences, which is a particular feature of 68 Ga-PSMA-11. Similar data were published by the same group in 2020 [20] regarding patients with a very early recurrence of disease (PSA < 0.5 ng/mL). In this latter paper, the authors reported an overall detection rate of 54.5% (similar to our findings with FCH PET/MRI), although they included patients with ongoing HT, and the most common site of PSMA-positive recurrence was in the lymph nodes.

As already stated by Freitag et al. [21], MRI outperforms FCH and 68 Ga-PSMA-11 PET in detecting local recurrences. In our study, the overall detection rate was 65.7% for mpMRI, irrespective of the site of disease, and therefore higher than for FCH PET alone (37.1%). This goes to show the added value of mpMRI for the purpose of identifying local recurrences, which would be lost using a PET/CT scanner. In accordance with Beheshti et al. [22], the local recurrence detection rate based on FCH PET/CT was 38%, while in the present study we found a rate of 63% for PET/MRI in the same site of recurrence. The introduction of 68 Ga-PSMA-11 PET/MRI would be the best choice, however, because of the complementary and complete information provided by mpMRI and PET for the purpose of identifying local and lymph node recurrences.

In the present study, we found that FCH PET/MRI prompted changes to RT planning in 36% of cases, more than when FCH PET/CT was used (28.5% and 21.6% in the reports from Wurschmidt et al. [23] and Alongi et al. [24]). We also found that the choice of treatment guided by FCH PET/MRI was appropriate in 74% of all patients, with a significant effect on subsequent PSA levels. The gain in terms of treatment appropriateness was associated mainly with a higher sensitivity of PET/MRI in detecting recurrences than that of MRI or PET alone (79% vs. 70% and 38%, respectively). To our knowledge, this is the first study correlating the results obtained with FCH PET/MRI with the effect on PSA levels after treatment.

In PCa, PET/MR can be performed by diverse radiopharmaceutical agents [25, 26]. A recent meta-analysis showed that PSMA PET/MR has a higher pooled detection rate for the identification of biochemical recurrence as compared to Choline PET/MR (81.8% vs. 77.3%, respectively), independently from the PSA range [26]. However, the highest difference in detection rate, between 18F-Choline and 68 Ga-PSMA PET/MR, was found for PSA level < 1 ng/mL (42.86% vs. 75%, respectively) [2, 27]. Until now, few data are now available about the role of Fluciclovine PET/MR in biochemical recurrence of PCa [25]. Moreover, to date, no head-to-head comparative data for all radiopharmaceutical agents and PET/MR are available.

The present study has some limitations to consider. First, there is the retrospective nature of the analysis and relatively small size of our sample, meaning that the results need to be confirmed in larger series. Although we only considered a small population, it was very carefully selected, as we only enrolled patients with a disease recurrence after RP, and without any ongoing HT. Second, there is the absence of any correlation between PET/MRI findings and PSA kinetic data (i.e., PSAdt and PSA velocity). Data for PSAdt were only available for 20 patients, whose values were lower in the case of a positive PET/MRI than in those with a negative scan (10 vs. 13 months), although the difference was not statistically significant. Achard et al. [11] likewise found no significant differences in PSAdt between positive and negative FCH PET/MRI.

In conclusion, FCH PET/MRI seems to achieve a high detection rate in patients with recurrent PCa and OMD, especially for PSA levels > 0.5 ng/mL. In patients with a very early disease recurrence (PSA < 0.5 ng/mL), it would probably be preferable to use 68 Ga-PSMA-11 PET/MRI. In our study, PET/MRI proved more sensitivity than MRI or PET alone, guiding appropriate therapeutic approaches in more than 70% of patients, as confirmed by their PSA levels.

### Patient summary

FCH PET/MRI could be useful in patients with a biochemical PCa recurrence and PSA levels higher than 0.5 ng/mL and can guide the therapeutic approach.

## Supplementary Information

Below is the link to the electronic supplementary material.

Supplementary file1 (DOCX 14 kb)

Supplementary file2 (DOCX 16 kb)

## Abbreviations

BCR: Biochemical recurrences
CT: Computed tomography
DWI: Diffusion-weighted imaging
EBRT: External-beam radiotherapy
FCH: 18F-choline
GS: Gleason score
HT: Hormonal therapy
MRI: Magnetic resonance imaging
mpMRI: Multiparametric MRI
MRAC: MR-based Attenuation correction maps
OMD: Oligometastatic disease
PCa: Prostate cancer
PET/MRI: Positron emission tomography–magnetic resonance imaging
PSA: Prostate-specific antigen
PSAdt: Short PSA doubling time
PSMA: Prostate-specific membrane antigen
RP: Prostatectomy
RT: Radiotherapy
