# Supplementary material for: Comparison of MRI, PET, and 18F-choline PET/MRI in patients with oligometastatic recurrent prostate cancer
Source: Abdom Radiol (NY). 2021 May 28;46(9):4401–9. doi: 10.1007/s00261-021-03131-7 (PMC8346454; doi:10.1007/s00261-021-03131-7)
Supplement: Supplementary file 1 — Supplementary file1 (DOCX 14 kb) [file 261_2021_3131_MOESM1_ESM.docx]

**Table 1s.** Distribution of imaging results based on the clinical variables and follow-up data

| **PSA value** | **MRI** | | **PET** | | **PET/MRI** | |
| --- | --- | --- | --- | --- | --- | --- |
|  | **Negative (n=24)** | **Positive (n=46)** | **Negative**  **(n=44)** | **Positive**  **(n=26)** | **Negative**  **(n=18)** | **Positive**  **(n=52)** |
| < 0.5 ng/mL  0.5-1.0 ng/mL  1.1-2.0 ng/mL  >2.1 ng/mL | 16 (44)  7 (30)  1 (17)  0 | 20 (56)  16 (70)  5 (83)  5 (100) | 25 (69)  12 (52)  5 (83)  2 (40) | 11 (31)  11 (48)  1 (17)  3 (60) | 15 (42)  2 (9)  1 (17)  0 | 21 (58)  21 (91)  5 (83)  5 (100) |
| GS <= 6  GS = 7  GS > 7 | 2(18)  8(32)  14(47) | 9(82)  17(68)  16(53) | 7(64)  16(64)  18(60) | 4(36)  9(36)  12(40) | 2(18)  4(16)  12(40) | 9(82)  21(84)  18(60) |
| No PSA decline  PSA decline  Stable PSA* | 8(42)  14(33)  2(25) | 11(58)  29(67)  6(75) | 11(58)  28(65)  5(63) | 8(42)  15(35)  3(37) | 5(26)  11(26)  2(25) | 14(74)  32(74)  6(75) |

The results are expressed as number (percentage); GS= Gleason Score; PSA= prostate specific antigen; *variation in PSA value between 0 and 0.1 ng/ml
