# Supplementary material for: Comparison of MRI, PET, and 18F-choline PET/MRI in patients with oligometastatic recurrent prostate cancer
Source: Abdom Radiol (NY). 2021 May 28;46(9):4401–9. doi: 10.1007/s00261-021-03131-7 (PMC8346454; doi:10.1007/s00261-021-03131-7)
Supplement: Supplementary file 2 — Supplementary file2 (DOCX 16 kb) [file 261_2021_3131_MOESM2_ESM.docx]

**Table 2s.** Change of radiotherapy planning in 22 patients

| **Patient No.** | **Age** | **Planning RT** | **RT dosage** |
| --- | --- | --- | --- |
| 1 | 77 | Prostatic bed | 74 Gy / 37 fr |
| 2 | 70 | Lymph node | SBRT 35 Gy / 5 fr |
| 3 | 75 | Prostatic bed and Lymph node | 70 Gy / 35 fr / SBRT 30 Gy / 5 fr |
| 4 | 59 | Prostatic bed | HYPO 66 Gy / 30 fr |
| 5 | 69 | Lymph node | SBRT 35 Gy / 5 fr |
| 6 | 75 | Prostatic bed | 74 Gy / 37 fr |
| 7 | 64 | Prostatic bed and Lymph node | HYPO +SIB 66 Gy / 30 fr + 54 Gy / 30 fr |
| 8 | 70 | Prostatic bed | HYPO +SIB 66 Gy / 30 fr + boost 70.5 Gy |
| 9 | 77 | Prostatic bed | 70 Gy / 35 fr |
| 10 | 71 | Prostatic bed | 70 Gy / 35 fr |
| 11 | 81 | Prostatic bed | HYPO 66 Gy / 30 fr |
| 12 | 76 | Prostatic bed | HYPO 66 Gy / 30 fr |
| 13 | 61 | Prostatic bed and Lymph node | HYPO +SIB 61.6 Gy / 28 fr + 50.4 Gy / 28 fr |
| 14 | 63 | Prostatic bed | 70 Gy / 35 fr |
| 15 | 71 | Prostatic bed | HYPO +SIB 66 Gy / 30 fr + boost 70.5 Gy |
| 16 | 76 | Prostatic bed | 70 Gy / 35 fr |
| 17 | 63 | Prostatic bed | 70 Gy / 35 fr |
| 18 | 76 | Prostatic bed | 70 Gy / 35 fr |
| 19 | 54 | Prostatic bed | 70 Gy / 35 fr |
| 20 | 70 | Lymph node | SBRT 27 Gy / 3 fr |
| 21 | 59 | Prostatic bed | 70 Gy / 35 fr |
| 22 | 74 | Prostatic bed | HYPO 66 Gy / 30 fr |

Gy=gray; fr=fractions; SBRT=Stereotactic Body Radiation Therapy; HYPO= hypofractioned radiotherapy; SIB=simultaneous integrated boost;
